# GM-DETR: Research on a Defect Detection Method Based on Improved DETR

**DOI:** 10.3390/s24113610

**Published:** 2024-06-03

**Authors:** Xin Liu, Xudong Yang, Lianhe Shao, Xihan Wang, Quanli Gao, Hongbo Shi

**Affiliations:** 1 School of Computer Science, State and Local Joint Engineering Research Center for Advanced Networking & Intelligent Information Services, Xi’an Polytechnic University, Xi’an 710048, China; 220711015@stu.xpu.edu.cn (X.L.); 20230201@xpu.edu.net (X.Y.); xihan_wang@xpu.edu.cn (X.W.); 2 Shaanxi Province Institute of Water Resources and Electric Power Investigation and Design, Xi’an 710001, China; shihongbo@slbsxy.com

**Keywords:** transformer, DETR, GAM, defect detection

## Abstract

Defect detection is an indispensable part of the industrial intelligence process. The introduction of the DETR model marked the successful application of a transformer for defect detection, achieving true end-to-end detection. However, due to the complexity of defective backgrounds, low resolutions can lead to a lack of image detail control and slow convergence of the DETR model. To address these issues, we proposed a defect detection method based on an improved DETR model, called the GM-DETR. We optimized the DETR model by integrating GAM global attention with CNN feature extraction and matching features. This optimization process reduces the defect information diffusion and enhances the global feature interaction, improving the neural network’s performance and ability to recognize target defects in complex backgrounds. Next, to filter out unnecessary model parameters, we proposed a layer pruning strategy to optimize the decoding layer, thereby reducing the model’s parameter count. In addition, to address the issue of poor sensitivity of the original loss function to small differences in defect targets, we replaced the L1 loss in the original loss function with MSE loss to accelerate the network’s convergence speed and improve the model’s recognition accuracy. We conducted experiments on a dataset of road pothole defects to further validate the effectiveness of the GM-DETR model. The results demonstrate that the improved model exhibits better performance, with an increase in average precision of 4.9% (mAP@0.5), while reducing the parameter count by 12.9%.

## 1. Introduction

The aim of defect detection [1] is discovering the appearance defects in various industrial products, agricultural products, and construction roads. It is one of the important technologies used to ensure product quality and maintain production stability. In recent years, the development of the manufacturing industry has gradually improved the requirements for industrial product quality inspection, while accurate defect detection results not only ensure that industrial products meet the necessary quality standards but also reduce the possibility of safety hazards in the use of industrial products. The advancement of the manufacturing industry has raised the standards for quality inspections of industrial products. Accurate defect detection not only guarantees product quality but also minimizes safety risks during product use [2]. However, defect detection is extremely difficult due to issues with complex background interference, low resolutions, and varying scales of defects. Currently, deep learning is the most commonly used technique for defect detection, and several image processing methods have been developed [3]. Traditional defect detection methods typically rely on handcrafted features tailored to specific types of targets, necessitating the design of numerous parameters for different target types. With the continuous development of computer technology, deep-learning-based machine vision methods have been increasingly researched and applied. Compared to traditional methods, deep learning methods can automatically extract effective features from input images without the need for manually designing complex features. The classical object detection networks include the SSD [4], Faster R-CNN [5], and YOLO [6] series, among others. These networks have become mainstream in the field of object detection due to their efficient detection methods. For example, Li et al. [7] used YOLO to detect rolling steel, achieving detection accuracy rates of up to 99% and a speed of 83 frames per second (fps). This trend has increased the application of deep learning algorithms in object detection. However, most defect detectors often suffer from complex detection pipelines and suboptimal performance due to their overreliance on handcrafted components such as anchors, rule-based target assignment, and non-maximum suppression (NMS). The recently proposed detection transformer (DETR) [8] eliminates the need for such handcrafted components and establishes a fully end-to-end framework for object detection. It uniquely utilizes self-attention instead of convolution, which overcomes the limitations of convolution methods that restrict the receptive field size. This often requires multiple layers to attend to the entire feature map [9]. Self-attention, on the other hand, can be used to capture global spatial information for the feature map through simple queries and assignments. This approach focuses more on the global characteristics of the targets, improving its ability to detect large defects. Additionally, the DETR treats the object detection problem as a set prediction problem and uses bipartite graph matching for label assignment [10]. This approach changes the object detection process from predicting multiple individual objects to treating all detected objects as a whole, enhancing the model’s global attention to the targets. In conclusion, the DETR leverages its unique mechanism to excel in handling global information, showing great potential to become a new approach for defect detection.

While the DETR has unique advantages, it also suffers from drawbacks such as a lack of control over the details of defective images [10], slow convergence speeds, and other issues. During initialization, each query in the transformer assigns almost the same weight to all positions, requiring the network to undergo a long training period to focus the self-attention feature on specific regions. To mitigate the consumption caused by the large-scale feature map input to the decoder, the DETR employs a downsampling strategy. However, this results in the loss of detailed information, leading to suboptimal performance in detection tasks. The existing research is focused on improving the feature extraction and feature fusion processes [11]. Some scholars have enhanced the adaptability of sample features for various scenarios by introducing adaptive mechanisms or incorporating transfer learning [12]. Others have adjusted the extracted features by introducing upward or downward paths and modifying the attention during the fusion process to process more relevant information.

In contrast, our core idea is to maintain the original framework’s focus on local feature information without modifying the self-attention mechanism, we introduce additional GAM attention to assign appropriate weights to small targets. The fused features not only capture enough local information, but also emphasize detailed information, aiding in optimizing the model’s performance in detecting target defects. However, while enhancing feature fusion, this may lead to an increase in the original network’s parameter count. Secondly, we have found that the original network’s loss function maps differences to [0, 1] using the logistic function, which may emphasize subtle differences in samples. Thus, during training, this may lead to the problem of not fitting to the optimal solution, thereby affecting the detection and fitting speed of samples with small differences. To address these issues, our research has three main contributions:

- This paper proposes combining the global attention (GAM) with the self-attention of the transformer. Integrating global and local information allows the network to more distinctly distinguish defects from the background. The recalibration of local features reduces information diffusion and amplifies interactions between global features, thereby enhancing the neural network's performance and improving defect detection.
- In this paper, to prevent the excessive number of model parameters from increasing the computational cost, we need to filter out unnecessary model parameters. We propose a layer pruning strategy to optimize the decoding layer, thus reducing the number of model parameters.
- This paper addresses the issue of the original loss function’s poor sensitivity to small differences in targets by replacing it with the MSE loss. Due to its simplicity, the MSE loss is faster and more sensitive to small differences in targets, allowing for better differentiation. This change not only improves model accuracy but also enhances convergence speed.

The rest of the paper is organized as follows. Section 2 summarizes the current related work; Section 3 introduces the methods used in this paper and the specific improvements made to the model; in Section 4, we have performed data augmentation to enhance the model’s robustness, explaining and discussing the experimental results; Section 5 concludes the research presented in this paper.

## 2. Related Work

This section discusses two main aspects of defect detection, which are categorized into traditional defect detection methods and deep learning methods [13]. Conventional defect detection methods usually use hand-designed characteristics to adapt to specific types of targets. This approach requires a large number of parameters to be designed for different target types. Researchers have proposed several methods based on these structural characteristics, including automated statistical methods, model-based methods, and frequency domain methods. Statistical methods usually divide images into blocks [14]. The target features are extracted by analyzing the gray value distribution of the target pixels. Commonly used methods include autocorrelation measures, co-occurrence matrices, and variance averaging. But these methods have a limitation; they struggle to distinguish between the blurring of the mean gray level and small targets. Anitha and Radha [15] used an independent component analysis algorithm to extract the required features and structural information from the image data. The structural information can be reduced by phase coherence. This allows the template image to be distinguished from the input image. However, these methods are not satisfactory for detecting defects in complex images. The modeling approach treats the structural characteristics of the target defects as a stochastic process and utilizes statistical information. The main methods include Gaussian Markov Random Field Models and Gaussian Mixture Models [16]. These methods can detect target defects, but may cause false negatives or false positives when facing complex backgrounds and small targets because small defects will merge with the background noise. Statistical detection of these defects is challenging. Additionally, surfaces defects can be detected based on their characteristic textures. This detection can be achieved by converting signals from the time domain to the frequency domain. Commonly used methods for defect detection include the Fourier transform and the wavelet transform, which suppress certain waveforms to identify defects. Specifically, defects can be detected through changes in these waveforms analyzed by these transforms. For example, Zalama et al. [17] proposed a solution based on instrumented vehicles using the Gabor filter approach to detect road defects such as longitudinal and transverse cracks. Their method has shown promising results compared to other methods. However, their high redundancy and computational cost relative to deep learning algorithms may make it less efficient for detection.

Defect detection algorithms have rapidly developed, resulting in an increase in proposed algorithms as a result of the gradual transition from traditional methods to deep learning. Detectors used for target detection tasks can be categorized into two main classes: one-stage and two-stage detectors. The two-stage detection framework uses a region proposal network (RPN) to generate region proposals, which are then subjected to region prediction. Common two-stage detectors include R-CNN, Faster R-CNN, and mask R-CNN [18]. In contrast, the one-stage detection framework integrates candidate frame extraction, CNN feature learning, and non-maximum suppression (NMS) into a single process. It outputs the location and classification of the target. Common one-stage detectors include SSD, YOLO, Retina Net [19], and FCOS [20]. This approach is becoming mainstream. For example, H Xie et al. [21] proposed an improved fabric defect detection algorithm based on SSD. They enhanced the traditional network structure by adding an FCSE module, which improved the network’s detection accuracy and verified the algorithm’s effectiveness. B Hu et al. [22] proposed a deep learning-based image detection method for PCB defect detection which utilizes Faster R-CNN, an architecture enhanced with the residual units of ShuffleNetV2. Experimental results indicate that the method is more suitable for use in production than other PCB defect detection methods. However, its effectiveness diminishes in complex scenes. Bing Hu et al. [23] utilized a two-stage industrial defect detection framework with YOLOV5 and Optimized Inception ResnetV2 to accomplish localization and classification tasks using two specific models. It was verified that the superiority and adaptability of the two-stage framework reached 91.0% in the constructed industrial defect environment. But the detection speed could not reach the actual demand. Xun Cheng et al. [24] proposed a new deep neural network, Retina Net, for steel surface defect detection. This network has shown better detection results compared to other networks, but it was only verified for defective targets in a simple dataset. Overall, both one-stage and two-stage defect detection algorithms have yielded improved detection results in the industry.

Most of the approaches discussed still rely on many manual components, such as anchor generation, rule-based training target assignment, and non-maximal suppression (NMS) post-processing. Therefore, they are not considered end-to-end. In recent years, a self-attention-based method called DETR has been proposed. Unlike the detectors mentioned earlier, this approach utilizes the transformer encoder–decoder architecture to achieve object detection tasks through full attention connections. This architecture eliminates many manual design elements in traditional object detection methods, thereby making the model more concise and efficient. The DETR method has achieved end-to-end object detection, thus more effectively emphasizing the holistic and integrated characteristics of the model. 

However, while DETR aims for model integration, it lacks control over the details of the detection targets. This oversight can lead to missed or incorrect detections, particularly in complex scenarios, thereby compromising the accuracy and stability of the detection results. To address these issues, Wang [25] and others recently proposed applying a query-based anchor point design, a technique widely used in CNN-based methods, to DETR. By doing so, this approach effectively resolves the challenge of accurately detecting multiple targets within a single region. Dai et al. [11] introduced dynamic attention in both the encoder and decoder stages of DETR to overcome limitations related to small feature resolution and slow training convergence. Li et al. [26] introduced a self-attention up-sampling (SAU) module to effectively capture features of small objects. Deformable DETR [27] proposes fusing the sparse spatial sampling of deformable convolutions with the transformer’s capability to model correlations across the entire feature map, reducing training time and enhancing defect detection efficiency.

## 3. Methods

In this section, we first review the fundamental architecture of DETR and then introduce the proposed improved GM-DETR framework. We also illustrate how our method more effectively captures the diversity among defect samples, thereby enhancing detection efficiency. Finally, we demonstrate its effectiveness on a dataset comprising 3390 real road pothole defect images.

### 3.1. The DETR Architecture

DETR is an end-to-end object detector based on the transformer architecture, designed to simplify the typical object detection pipeline by eliminating several manually designed components. Additionally, the model further simplifies the detection process by treating it as a set prediction problem. The model adopts a transformer [28] encoder–decoder architecture, where the self-attention mechanism explicitly models all pairwise interactions among elements in the sequence. This detailed modeling enables the system to effectively address the issue of redundant predictions. Unlike previous detection methods, DETR employs a dichotomous matching loss function to accurately align predicted results with real objects. The ordering of predicted objects is fixed, allowing the model to parallelize its output generation without requiring sequential generation. DETR directly performs set predictions, which ensures a unique match between predicted and actual bounding boxes [28]. Once a successful match is achieved, the model calculates loss values for several attributes of the predicted box, including its score, category, center coordinates, and dimensions. DETR’s distinctive features include the absence of custom layers, the use of a dichotomous matching loss function, and the combination of transformers with parallel decoding. These characteristics make it an efficient and easily replicable object detection algorithm that can be adapted to other frameworks.

**Figure 1:**
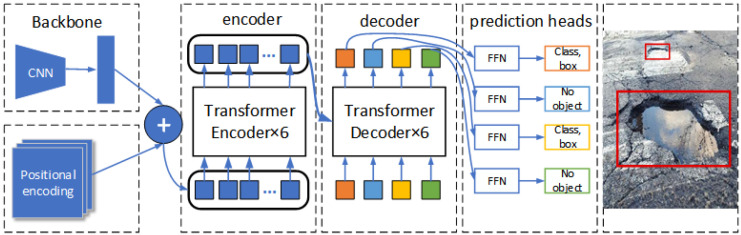
The overall architecture of DETR, where the red boxes are detected targets.

The simplified overall architecture of DETR is illustrated in Figure 1, which includes three main components: the CNN backbone network diagram, the encoder–decoder structure, and a simple feed-forward network. In this case, the CNN backbone consists of multiple convolutional and pooling layers. The CNN backbone is primarily responsible for two key functions: extracting features from the input image to generate a lower-resolution feature map and incorporating positional coding to preserve the positional information. In contrast to the original transformer architecture, the transformer encoder in DETR utilizes the self-attention mechanism to process a fixed number of learned positional embeddings. The decoder module simultaneously processes all object queries without masking any, thus maintaining the contextual relationships across the entire image. This approach allows the transformer decoder to generate a higher resolution and richer feature representation. This improves the accuracy of object detection while preserving more details in the spatial dimension.

The process involves computing the set prediction loss, utilizing dichotomous graph matching [29] to determine the optimal match. It consists of the calculation of the ensemble prediction loss; in the process of loss calculation, the dichotomous graph matching [29] method is used to determine the optimal matching, which produces the best bipartite matching between the predicted object and the real object. To find the category labels and bounding boxes of the optimal prediction, the matching loss based on the Hungarian algorithm [30] can be described as in Equation (1):

(1) $$L_{Hungarian}\left(y,\hat{y}\right)=\sum_{i=1}^{N}\left[-1\log_{{\hat{p}}_{\hat{\sigma (i)}}}(c_{i})+1_{\left\{c_{i\neq \emptyset }\right\}}l_{box}\left(b_{i},{\hat{b}}_{\hat{\sigma }}(i)\right)\right]$$

where $1_{\left\{c_{i}=\emptyset \right\}}$ is a bool function that is 1 at that time and 0 otherwise. $c_{i}$ is the category label of the ith object, and $\sigma \left(i\right)$ is the index of the bounding box that matches the ith target. ${\hat{p}}_{\hat{\sigma (i)}}\left(c_{i}\right)$ denotes the probability that the category of the first predicted bounding box of the $\sigma \left(i\right)$ prediction of the DETR prediction is $c_{i}$. The second half of the loss is calculated for the bounding box $L_{box}\left(b_{i},{\hat{b}}_{\sigma \left(i\right)}\right)$; the method performs the box prediction directly [30], which simplifies the process to some extent. However, the L1 loss still scales differently for small and large bounding boxes, despite having similar relative errors. To address this problem, the DETR model uses a linear combination of the L1 loss and a generalized iou loss of $L_{iou}\left(b_{i},{\hat{b}}_{\sigma \left(i\right)}\right),$ which is scale-invariant and defined as shown in Equation (2):

(2) $$L_{box}\left(b_{i},{\hat{b}}_{\sigma \left(i\right)}\right)=\lambda_{iou}L_{iou}\left(b_{\sigma \left(i\right)},{\hat{b}}_{i},\right)+\lambda_{L1}{\left\|b_{\sigma \left(i\right)}-{\hat{b}}_{i}\right\|}_{1}$$

where $\lambda_{iou}$,$\lambda_{L1}\in R$ are hyperparameters and these two losses are normalized by the number of objects in the batch.

#### 3.1.1. Self-Attention

The self-attention mechanism functions as an aggregator, primarily building relationships within elements by computing the association between each element and all other elements in the context [31]. Furthermore, this mechanism enables the model to dynamically focus on various information positions when processing sequential data, unconstrained by a fixed window. In the DETR model, the image feature representation at each position is considered as an “element”, with correlations among these elements being calculated. Elements with higher correlations receive higher attention scores. These scores are transformed into weighted representations, which generate new representations for each position, thus aiding the model in decision making [32]. The computation of self-attention is defined as follows. Given d as the dimension of the embedding, n as the number of vectors, and $Χ\in R^{n\times d}$ representing the sequence of vectors, $W_{q},\;W_{k}$, $W_{v}$ is the learned weight matrix and for each position in an input sequence, the Query, Key, and Value vectors are derived by applying these matrices through a linear transformation, i.e., $Q=ΧW_{q},K=ΧW_{k},V=ΧW_{v}$, where $d_{k}$ denotes the dimension size of Q, K for scaling the attention size. The formula for calculating the attention score is provided in Equation (3):

(3) $$Attention\left(Q,K,V\right)=Softmax\left(\frac{Q\cdot K^{T}}{\sqrt{d_{k}}}\right)\cdot V$$

**Figure 2:**
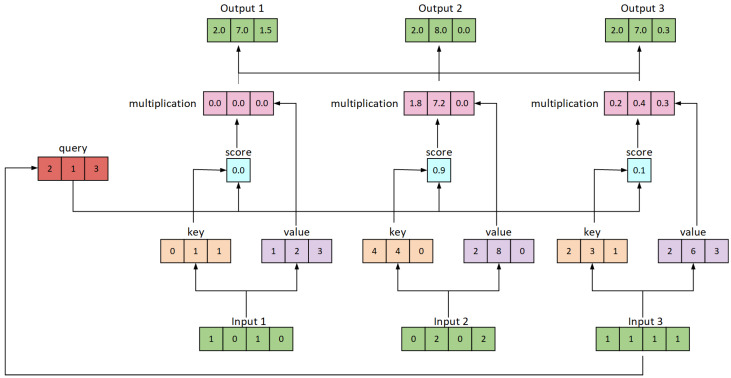
Self-attention mechanism.

The similarity between two vectors is calculated by the dot product of $Q\cdot K^{T}$ and divided by $\sqrt{d_{k}}$. Subsequently, this result is converted into a probability distribution using a softmax operation. Finally, the resulting values are matrix-multiplied with V to obtain the weighted sum, which represents the output for each position, as shown in Figure 2:

Initially, we input three vectors, each of dimension 4, corresponding to the Q (Query), K (Key), and V (Value). According to the diagram, each vector form has a dimension of 3. At this point, the weight matrix has a dimension of 4×3. We multiply these three input matrices by respective weight matrices to generate the Q matrix, and repeat this process by multiplying them by the weight matrices for K and V to obtain the corresponding K and V matrices. Next, we perform element-wise multiplication between the Q from input 1 and each K from the inputs, resulting in attention scores. We then apply a Softmax operation to these scores and update the original score vector. The softmaxed attention scores are used to multiply each V from the inputs to obtain weighted values. These values V are subsequently summed to produce output 1, as depicted in the figure. Finally, attention scores are calculated for inputs 2 and 3, respectively, and they are processed to obtain the final output 2 and output 3.

#### 3.1.2. Multi-Head Attention

In the self-attention mechanism, an attention head can only focus on specific locations in the element. Still, a single attention head may not adequately capture the global dependencies and interactions of internal features in the input sequence. For this reason, additional heads can be added to the mechanism, each responsible for focusing on different aspects of the element, a configuration referred to as multi-head attention [33]. By mapping elements to multiple independent attentions, attention weights for each position in the elements can be calculated in parallel. In this way, different heads can focus on distinct feature aspects. In DETR, multi-head attention is primarily used in the decoder phase to facilitate cross-attention between object queries and encoder features. This facilitates modeling the positions and contextual relationships of the targets more effectively. Specifically, the computation for multi-head attention heads is expressed as shown in Equation (4):

(4) $$MultiHead\left(Χ\right)=Concat\left({head}_{1}\ldots ,{head}_{h}\right)W^{\prime }$$

where h is the number of heads, ${head}_{i}$ denotes the ith attention head, and $W^{\prime }$ is the learnable weight matrix for projecting the output to the final multi-head attention value [34]. Each attention head is obtained by performing a self-attention computation on Q,K,V. The computation formula is defined as in Equation (5):

(5) $${head}_{i}=Attention\left(ΧQ_{i},ΧK_{i},ΧV_{i}\right)\;\;i=1,\ldots h$$

### 3.2. Improving the DETR Algorithm

**Figure 3:**
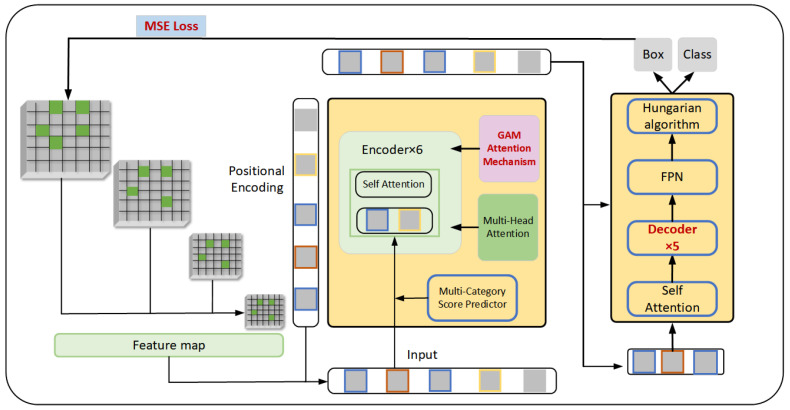
Structure of the GM-DETR model.

To better adapt to the variability among defect samples, this paper modifies the model structure to focus on local features in the image that are relevant to defects. This enhancement is intended to improve detection accuracy under the limitations posed by variations. Furthermore, to further improve detection precision, this study explores additional methods, including the application of anchor point designs, commonly used in CNNs to the DETR model. In defect detection, attention mechanisms are often used to enhance the model to better focus on the differences between the regions of interest and the background, improving the accuracy of object detection. After evaluating various attention mechanisms, this study selected the Global Attention Mechanism (GAM), which spans spatial channel dimensions, due to integration into the original DETR model. Integrating self-attention into the transformer along with global GAM attention enhances the modeling of spatial relationships between targets, facilitates consideration of global context information, and allows adaptation to various target sizes and quantities. However, these operations can increase the computational complexity of the model, leading to longer detection time and increased Frames Per Second (FPS) values. To address this issue, we propose a trade-off strategy: the layer pruning strategy, which aims to filter out unnecessary model parameters. This reduces the model’s complexity and eases training difficulty. Based on this, the above improvement may have the issue of slow convergence of the model during training. We apply the improved loss function to enhance the detection accuracy of the model further, and the specific improved model diagram is shown in Figure 3.

#### 3.2.1. Attention Optimization

In DETR, the input image is first fed into the ResNet50 network of CNN for feature extraction to obtain the feature layer. Simultaneously, the network encodes the position of the feature layer obtained here to gain more information about the position of the defects. These two feature layers are fused and spliced together and the fused feature layer is input into a coding network. This network utilizes a transformer structure for encoding and decoding the features and uses a self-attention mechanism to model the input sequences within the encoder. However, since the self-attention mechanism focuses on local feature information [35], it faces the problem of information loss and blurring when processing long sequences. This often results in insufficient contextual information for some targets, thus creating a situation where the control of image details is missing. At the same time, the design of self-attentive allows each position in the decoder to attend to every other position in the input sequence. However, smaller targets within complex contexts may be relatively sparse in the image and interact less with other objects, making it difficult for the model to accurately capture the features of these targets. Additionally, this model utilizes object query vectors to match the encoder features, which facilitates the retrieval of the location and category information for the target [35]. Nevertheless, the number of object query vectors is typically fixed and limited relative to the length of the input sequence. This means that smaller object queries may not receive sufficient attention in self-attention, making them difficult to detect. 

Attention mechanisms are implemented by adding different modules to the network and play a crucial role in deep learning. They enable neural networks to selectively focus on different parts of the input data, thus improving the model’s ability to capture essential information. In deep learning, specific types of attention modules, such as self-attention mechanisms and Global Attention Modules (GAMs) [36], are extensively employed in various tasks, including natural language processing, computer vision, and speech recognition. The primary goal of incorporating these attention mechanisms is to enhance the adaptability of models, enabling them to flexibly adjust their focus on input information flexibly across different contexts.

To address the issues mentioned, we propose fusing attention modules to enhance the capture of global and local contextual information in sequence data processing. This design aims to enhance the network’s ability to represent objects across different scales. With the introduction of the Global Contextual Attention module, our goal is to provide the model with robust global sensing capabilities, enabling more efficient capture of global correlational information [35]. Traditional neural network models often face limitations from local information when processing sequential data. The introduction of GAM aims to improve the model’s ability to recognize small targets by learning the importance of different positions in the input sequence. GAM attention calculates attention weights for each input element and considers the relationships among all elements. It can capture more prominent dimensional features and enhancing the model’s capacity to recognize small targets [36]. 

**Figure 4:**
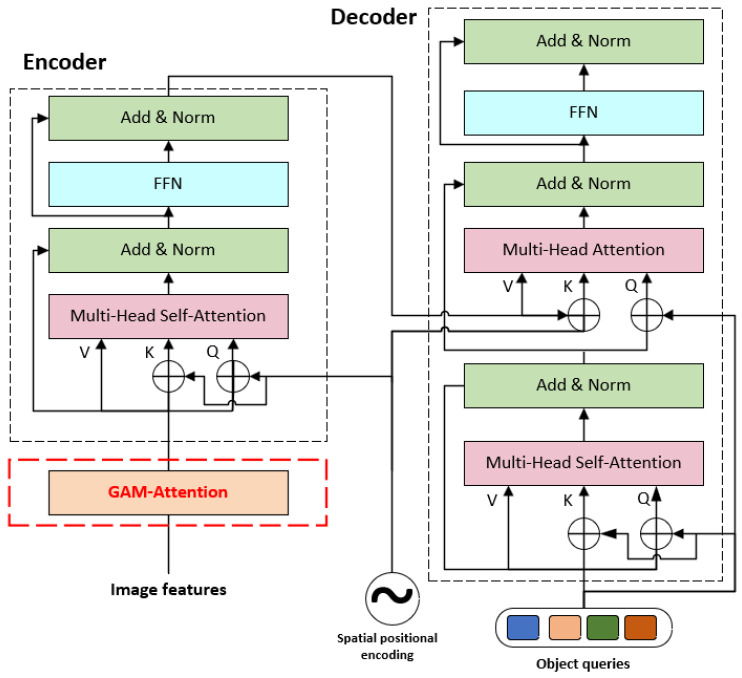
Improved network architecture.

In the network, we embedded the attention module after the backbone extraction network and the location information encoding module. This configuration is designed to enhance the extraction of feature information before the self-coding process and to improve the network’s fitting ability, as shown in Figure 4.

**Figure 5:**
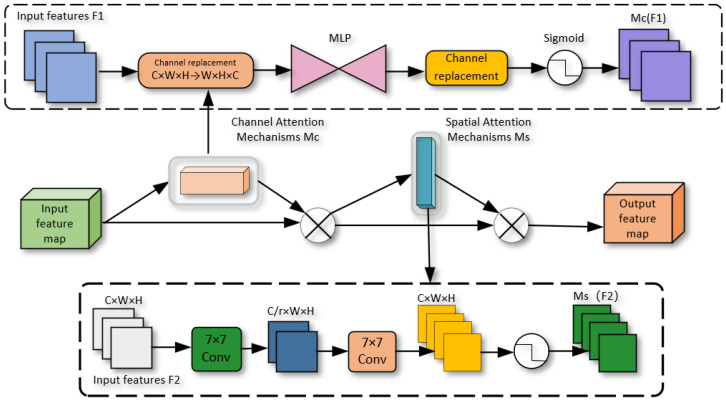
GAM attention mechanism.

Specifically, traditional channel attention typically employs Global Average Pooling (GAP) to calculate each channel weight, but the features derived from GAP lack feature diversity, which hinders capture of detailed input feature information in images. In contrast, the channel attention in this paper is shown in Figure 5. It preserves the three-dimensional information through a technique called 3D substitution and amplifies the cross-dimensional channel-space dependencies using a two-layer Multilayer Perceptron (MLP) to capture the rich input feature information. The corresponding computational formula is shown in Equation (6). Notably, the channel attention mechanism can lead to some loss of positional information in the image. For this reason, the spatial attention mechanism is introduced, with its computation detailed in Equation (7).

(6) $$F_{2}=M_{c}\left(F_{1}\right)\times F_{1}$$

(7) $$F_{3}=M_{s}\left(F_{2}\right)\times F_{2}$$

where $M_{c}$ and $M_{s}$ denote the channel attention mechanism and spatial attention mechanism, respectively. The structure of the GAM module includes feature map input and attention map output. During the embedding process, we first use the convolutional layer to extract features from the input image to obtain the feature map, where each location corresponds to a specific region in the image. The feature map is inputted to the GAM module, and the feature map is transformed into a global feature vector using global pooling operation [36]. Subsequently, the multilayer perceptual machine (MLP) processes these global feature vectors to generate an attention map, which matches the size of the original feature map. The attention map indicates the relative importance of each location, and the feature map is multiplied with the attention map to obtain a weighted feature map through the global attention mechanism. More important regions receive higher weights, while less important regions will be assigned lower weights. This process aims to strengthen the representation of significant regions, focusing the model more effectively on key regions.

The GAM attention mechanism is an enhancement of the CBAM [37], which also uses spatial attention and channel attention, but does so with different adjustments. GAM sequentially captures global correlation information through a continuous attention-enhancing network, integrating both CAM [38] and SAM [39]. The schematic diagram is shown in Figure 5. Initially, the input feature maps undergo a dimension conversion, and the converted feature maps are input to the MLP and then converted to the original dimensions. The output is processed by Sigmoid processing. In the case of SAM [39], the number of channels is initially reduced and then increased. The number of channels is reduced by convolution with a 7×7 kernel to decrease the computational effort. Following this, a subsequent convolution operation with a 7×7 kernel increases the channels’ consistency back to their original count to maintain consistency. Finally, the process goes through a Sigmoid output.

In the DETR model, each target interacts with the whole image during the encoding stage, and each target can utilize the information from the entire image to assist in defect detection. This interaction can establish a link between the target and other regions in the image to better model the correlation between the targets. Even when there is occlusion or interdependence among targets, the GAM is not only enough to guide the model to focus on target-to-target correlations through the global attention mechanism, but also reduces information diffusion and amplifies global interactions to increase the performance of neural networks.

Following the integration of the GAM attention mechanism, the network pays more attention to the defect information to be detected. This enhancement significantly improves the extraction of location information and allows the network to detect road defects more accurately. The model dynamically adjusts the attention weights based on the size and position of the targets, enhancing its capability to pinpoint defects across various scales. Moreover, GAM attention can reduce the interference of background information on defect detection, enabling the network to perform more precise detection.

#### 3.2.2. Optimization of Transformer Structure

To maintain computational efficiency while introducing the Global Attention Mechanism (GAM), we propose a layer-pruning strategy. Traditional DETR models utilize many self-attentive layers in both the encoder and decoder to process the input sequence and generate target bounding boxes [8]. While the encoding layer extracts features from the input image, the decoding layer transforms these features into object detection results. However, this multi-layered structure contributes to a large number of parameters and extensive computational demands, which negatively impact the model’s efficiency. We found through analysis that pruning the decoding layers not only simplifies the model’s structure but also decreases computational complexity. This is because the decoder layers contain redundant parameters compared to the encoder layers. To be specific, we retained the core layer in the model—the coding layer. This layer converts the input image into a series of feature vectors, and uses these feature vectors as input to the decoder [8]. Subsequently, after carefully analyzing the coding and decoding structures alongside the feature maps, we selected the most representative and information-dense coding layer for retention. This layer not only effectively extracts a series of feature vectors from the input image but also eliminates redundant information and reduces dimensionality using Global Average Pooling. Simultaneously, we prune the number of decoding layers, which reduces the model’s complexity and the risk of overfitting. When there is less training data or more data noise, it is important to ensure that the model can maintain good performance while reducing the number of layers.

At the same time, pruning the decoding layers not only simplifies the model structure but also reduces its computational complexity. We will also verify the impact of pruning these layers on the performance of the model through experiments and evaluations. Preliminary results prove that the layer-pruning strategy effectively balances computational efficiency with performance. This strategy reduces the computational complexity while still maintaining a higher accuracy of target detection. This approach provides a highly efficient and accurate solution in the case of limited computational resources.

#### 3.2.3. Optimization of the Loss Function

**Figure 6:**
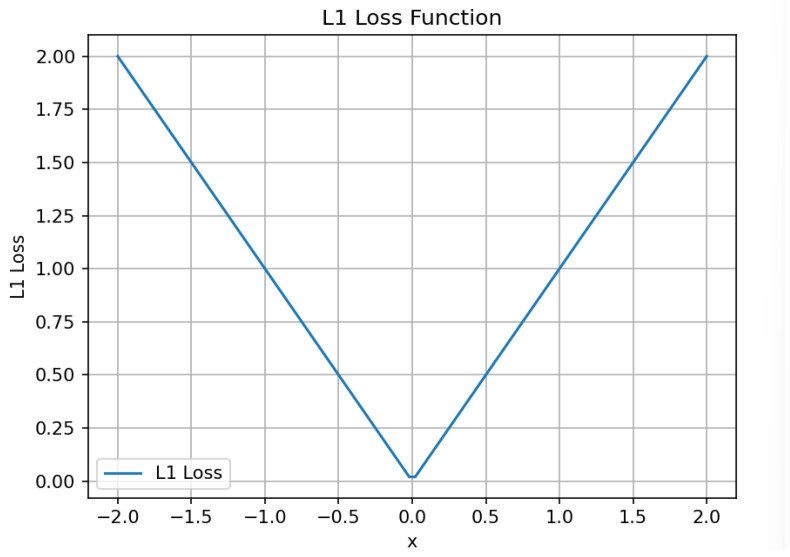
Schematic of the L1 loss function.

In this paper, the L1 loss [40] function previously used in the DETR model is replaced with the MSE loss function. DETR uses the L1 loss function to measure the difference between the predicted bounding box coordinate values and the true labeled bounding box coordinate values. Although the L1 loss function effectively measures the difference between the predicted and true values [40], it is susceptible to outliers because it calculates the sum of the absolute differences, as detailed in Equation (8), where $f\left(x_{i}\right)$ and $y_{i}$, respectively, represent the predicted value of the ith sample and the corresponding true value, and n is the number of samples; the curve distribution is shown in Figure 6. From the figure, it can be seen that the loss function is relatively sharp and not smooth, and it cannot be derived at zero, as the derivative of L1 is constant, so the smaller the value of the loss, the the larger the obtained gradient, which may result in the model’s oscillations being not conducive to convergence. Furthermore, we found that the L1 loss function maps the differences within the range [0, 1] through a logistic function. This approach tends to overemphasize samples with small differences. Therefore, it may lead to the problem of fitting less than the optimal solution during the training process, which in turn affects the detection and fitting speed for the samples with smaller differences.

(8) $$L1=\frac{\sum_{i=1}^{n}\left|f\left(x_{i}\right)-y_{i}\right|}{n}$$

**Figure 7:**
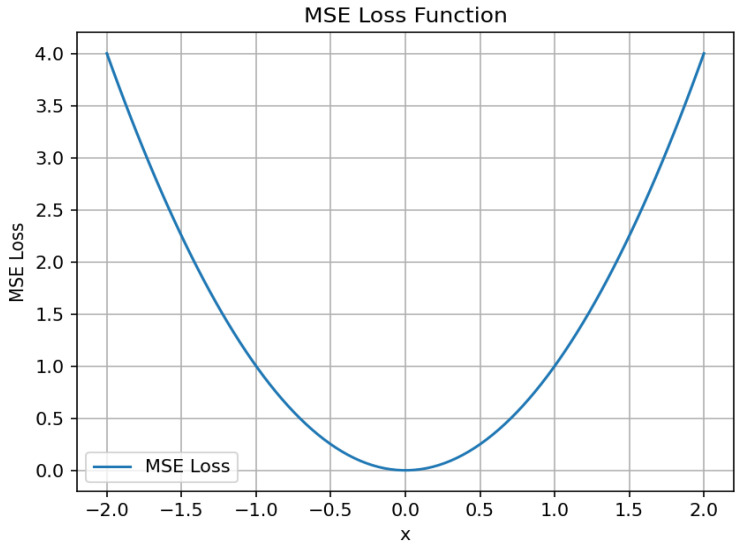
Schematic of the MSE loss function.

To address the above problem, we found that the MSE [41] is more sensitive to samples with smaller differences, allowing its ability to differentiate between them. This is because the MSE assigns larger gradient values to samples with smaller differences during gradient computation. Consequently, the model pays more attention to these samples during training and adjusts its parameters more quickly to minimize prediction errors. The specific formula of MSE is shown in Equation (9), where $f\left(x_{i}\right)$ and $y_{i}$ denote the predicted value and the corresponding true value of the ith sample, respectively, and n is the number of samples; the distribution of the curves is shown in Figure 7. We found that the function curves are continuous and can be guided everywhere. Notably, the gradient decreases as the value of error decreases, promoting convergence to the minimum value.

(9) $$MSE=\frac{\sum_{i=1}^{n}{\left(f\left(x_{i}\right)-y_{i}\right)}^{2}}{n}$$

To assess the effectiveness of the MSE loss function in target detection, we implemented the MSE loss function to replace the original L1 loss function in the DETR model. Concretely, we use the MSE loss function to measure the difference between the predicted bounding box coordinate values and actual bounding box during the training process [41]. Between the predicted and true values of each bounding box, the sum of the squares of the differences of the individual coordinates is calculated and then averaged over all dimensions. This approach not only increases sensitivity to smaller samples but also reduces the impact of outliers on model performance. Therefore, in regression tasks, the MSE loss pays more attention to larger errors during training and penalizes them more, which makes the model more inclined to reduce these large errors. Moreover, the MSE loss is computationally efficient, which further accelerates the model’s convergence speed.

We validate the DETR model using the MSE loss function on the pothole dataset and compared its performance with the traditional DETR model and other target detectors. The experimental results indicate that the DETR model using the MSE loss function achieves superior performance in target detection.

## 4. Experimental Results and Analysis

To verify the method’s effectiveness, we built an experimental platform using Windows 11.0 as the operating system and PyTorch 1.11.0 as the deep learning framework. We used DETR as the baseline network; Table 1 shows the experimental environment configuration.

We applied the same hyperparameters throughout the experimental training process, and Table 2 shows our parameter settings.

### 4.1. Datasets and Evaluation Indicators

**Figure 8:**
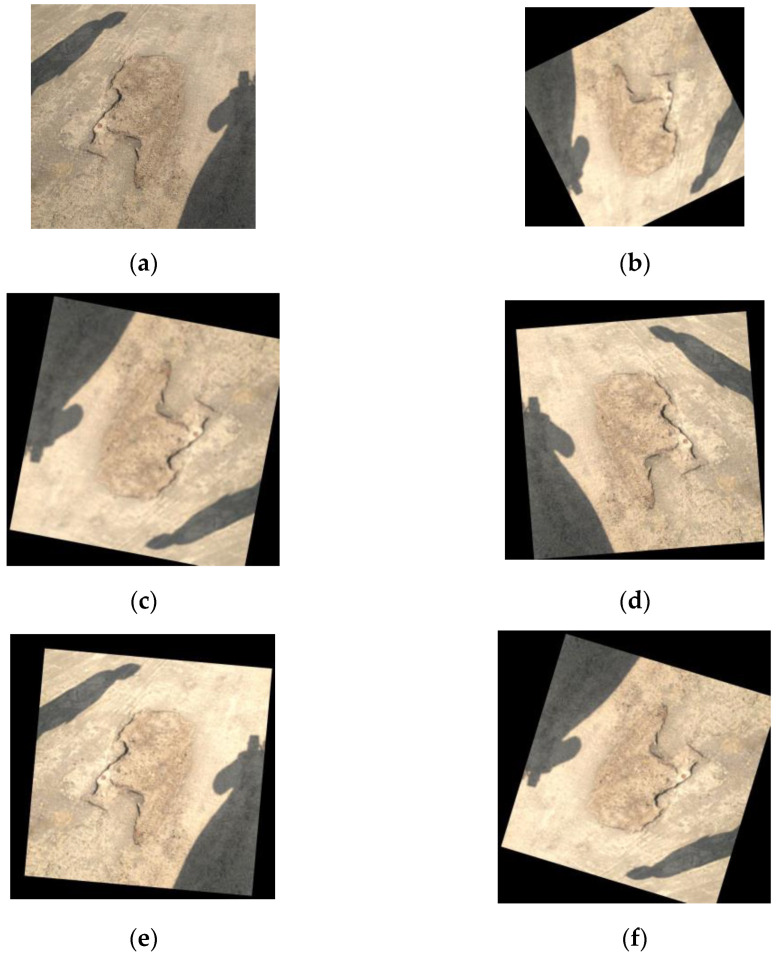
Examples of defective data enhancements: (**a**) original image; (**b**–**f**) the images generated after being randomly processed by various data augmentation algorithms.

In this study, we use a pothole dataset (Annotated Potholes Image Dataset) from a database managed by Kaggle to confirm our method’s efficacy, consisting of 665 images of real road potholes, each with a resolution of 720 × 720 pixels. Object classification was performed by a panel of experts by determining whether the pothole under study was present in the image and determining its location in the corresponding image. Since the small amount of data in this dataset causes the model to be overfitted, it is necessary to expand the dataset to improve the generalization ability and performance of the model. To provide an objective assessment of the performance of the defect detection model, after analyzing and processing, we performed data enhancement, which includes mirroring, inverting, increasing brightness, and adding noise to the original image, as shown in Figure 8. Using the method, we have expanded the dataset further. It currently comprises 3990 images that describe the detection targets of this study. The images are divided into the training set, validation set, and test set in the ratio of 8:1:1. We tested our method on these datasets. The primary evaluation metric, Frames Per Second (FPS), measures the detection speed of the model, indicating how many frames the system can process per second. Additionally, the size of the weight file created during the training of the neural network model is indirectly indicated by #Params (MB), which reflects the model’s complexity and resource demands. The performance of the target detector was evaluated by using four metrics (i.e., precision, recall, F1-score, and mAP).

Precision is defined as the proportion of true positive predictions out of all positive category predictions made by the model. Recall is the proportion of samples correctly predicted by the model to be in the positive category out of all samples that are actually in the positive category. Precision is the proportion of positive instances predicted by the model that is actually in the positive category. Mean Average Precision (mAP) is used to evaluate the model’s accuracy and is computed using the formula below. F1-score is the weighted average of Precision and Recall. These metrics collectively assess the performance and stability of the model.

The formulas contained therein are shown In (10)–(13) below:

(10) $$Precision=\frac{TP}{TP+FP}$$

(11) $$Recall=\frac{TP}{TP+FN}$$

(12) $$mAP=\frac{1}{N}\sum_{i=1}^{N}AP_{i}$$

(13) $$F1=2*\frac{Precision*Recall}{Precision+Recall}$$

**Figure 9:**
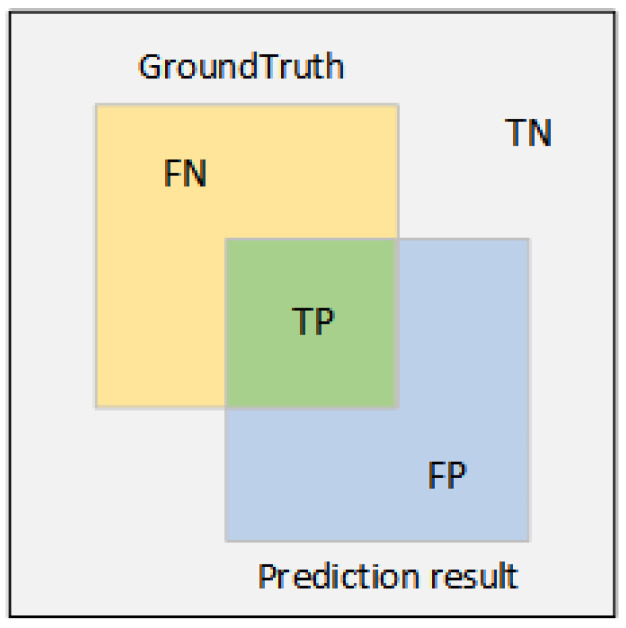
Sample relationship chart.

True Positive (TP) refers to the number of samples that are actually positive and predicted as positive by the model. True Negatives (TN) refer to the number of samples that are actually negative and are correctly predicted as negative by the model. Conversely, False Negative (FN) is the number of samples that are actually positive but predicted as negative by the model. False Positive (FP) is the number of samples that are actually negative but predicted as positive by the model. A more visual representation of the above relationships can be seen In Figure 9.

### 4.2. Experimental Results and Analysis

#### 4.2.1. Ablation Experiment

**Figure 10:**
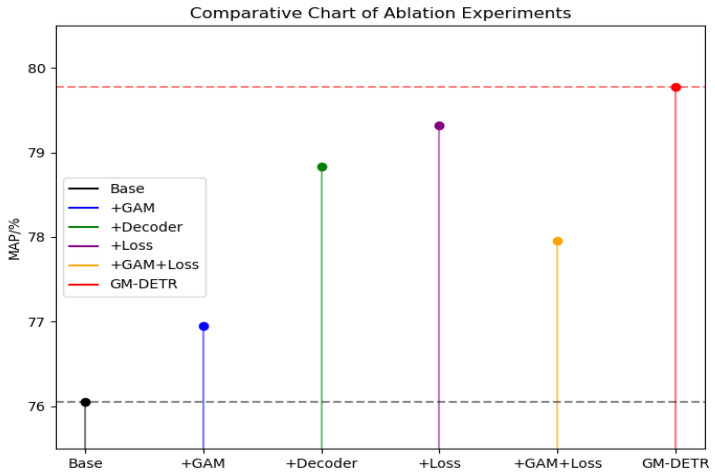
Ablation experiment figure.

Data from Table 3 and Figure 10 indicate that embedding the GAM attention after the backbone extraction network and location information encoding module slightly increases the number of parameters and computational complexity but yields a 1.2% improvement in accuracy. Secondly, our experiments showed that reducing the number of decoding layers decreased parameters by 13.4%, and there was a 3.6% improvement in the accuracy of the model. Therefore, the introduction of the MSE loss function improved the accuracy by 4.3%. Combining the loss function module with the GAM attention module improves the accuracy of the model. Although it increases the number of parameters somewhat, the improvement of DETR by combining the GAM, Decoder, Reduction and the MSE loss functions outperforms the original the DETR model in terms of detection accuracy. As demonstrated in Figure 8, on the same dataset, the loss in the validation set decreases to approximately 0.55 from the original 0.8. Consequently, the GM-DETR model achieves a 4.9% higher accuracy and a 12.9% reduction in the number of parameters compared to the original DETR model. In response to DETR’s original lack of control of image details, a significant improvement can be seen in the figure example.

Due to the GAM (Global Attention Map) mechanism, if there are defects or particularly important areas in the input image data, the model can better capture the key information in the input data to deal with the complexity of the defects in the input data. It can also assign greater weight to different features in the final inference and classification stage. This approach not only improves the detection of complex defects but also focuses more on the detection in the regions of interest.

**Figure 11:**
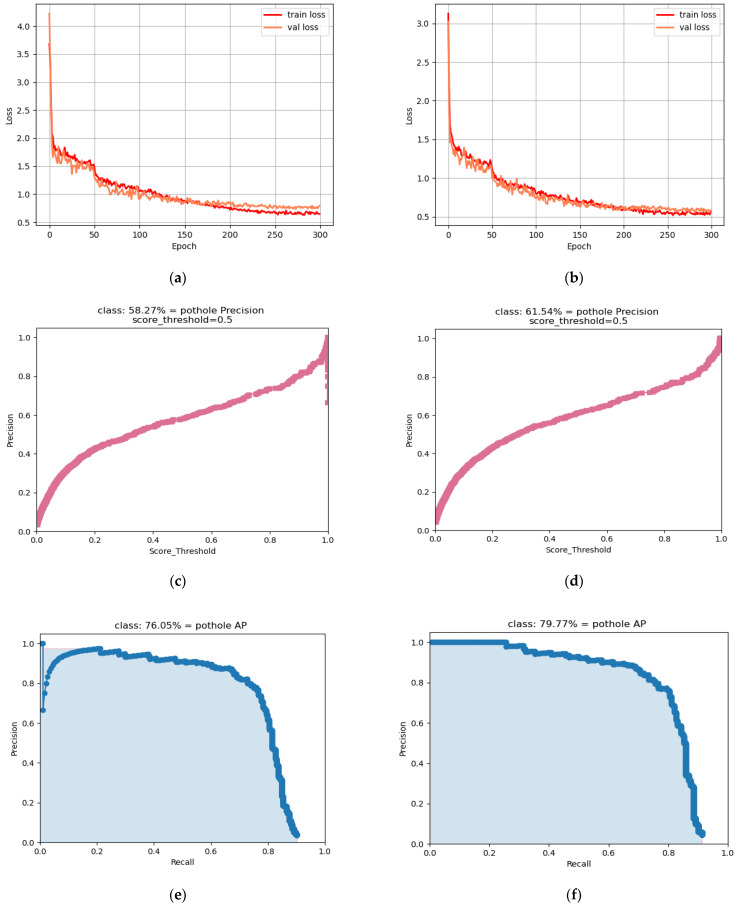
Evaluation with the original model: (**a**) loss graph for the DETR model; (**b**) loss graph for the GM-DETR model; (**c**) precision graph for the DETR model; (**d**) precision graph for the GM-DETR model; (**e**) precision and recall graphs for the DETR model; (**f**) precision and recall graphs for the GM-DETR model.

As shown in Figure 11 below, we can see that the loss curve training and validation sets are more closely matched to the smoothness. Additionally, the loss values for GM-DETR decrease rapidly within the same epoch and produce a relatively low stabilization value. Furthermore, it can be seen that the GM-DETR model converges faster. Moreover, on the Precision curve, more points are in the position where Precision and Recall are higher at the same time, indicating that the model’s prediction results contain most of the true positive examples. This performance represents points with excellent model performance, thus further validating that our method improves the accuracy of the model.

#### 4.2.2. Comparison of Different Attention Mechanisms

**Figure 12:**
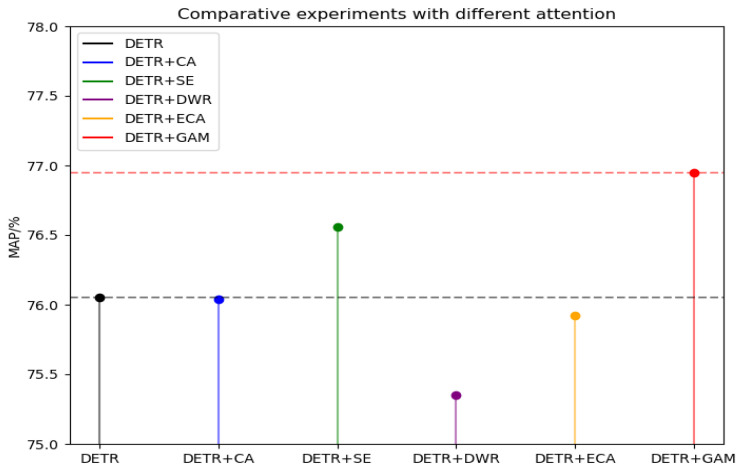
Comparative experiments with different attentions.

By embedding different attentions after the backbone extraction network and after the location information encoding module, the effect on the detection accuracy of the DETR model is shown in Table 4 and Figure 12. From the table, we can see that using different attentions has a different effect on the accuracy and the number of parameters, increasing in both the number of parameters and the accuracy. We utilized a combination of attention types, including Channel Attention (CA) [42], Squeeze and Excitation (SE) [43], Dynamic Weighted Residual (DWR) [44], Efficient Channel Attention (ECA) [45], and Global Attention Module (GAM) [36]. In some cases, we can see that the use of attention decreases the accuracy, but the accuracy improves when using channel attention CA, SE, and the global attention GAM. This underscores that channel attention significantly enhances network accuracy. However, global attention demonstrated a more pronounced effect, boosting model accuracy to 76.95%, which is enough to better compensate for the defects of self-attention in DETR, and has better detection accuracy compared with channel attention CA and SE.

#### 4.2.3. Performance Comparison of Different Models

**Figure 13:**
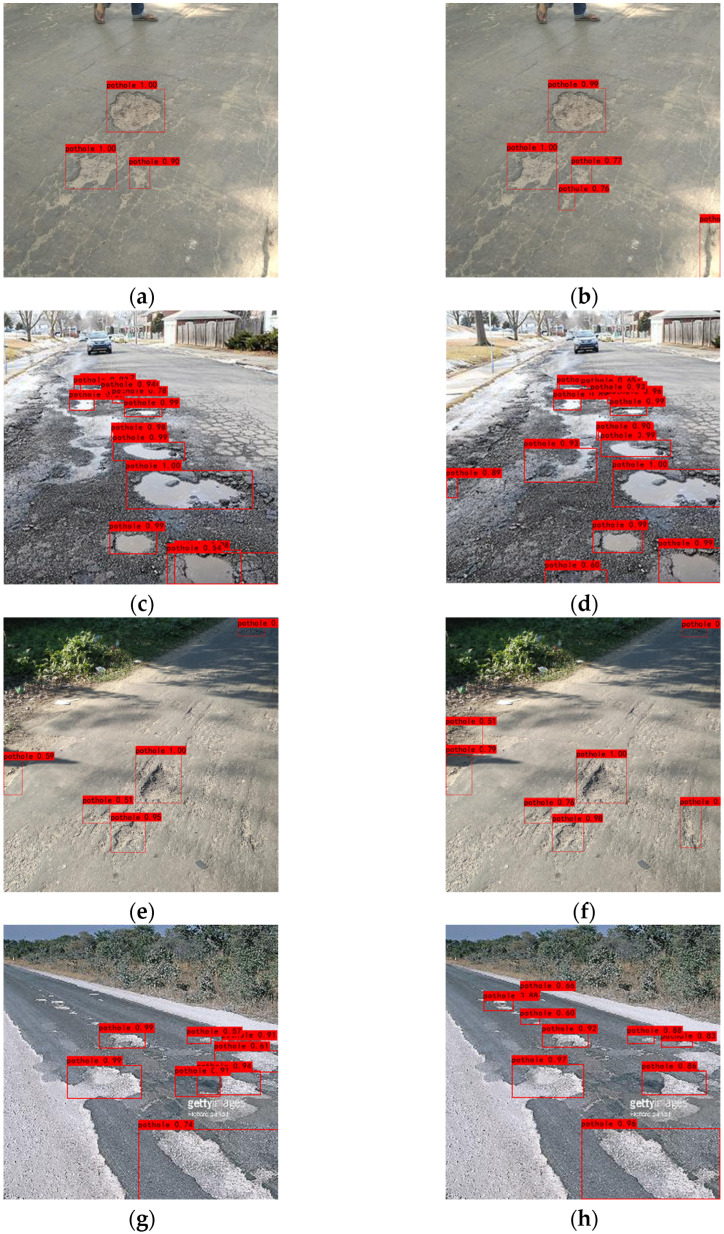
Before and after the improvement of this graph: (**a**,**b**) the first group; (**c**,**d**) the second group; (**e**,**f**) the third group; (**g**,**h**) the fourth group.

Figure 13 shows the detection results of the network on the dataset. Comparative analysis reveals that our algorithm can improve the problem of missing control of image details and improve the detection of smaller defects. In the graph of detection results, we can see that the detection in complex backgrounds has been significantly improved, and the problem of missed detection has also been improved. This is mainly because we combine the global attention GAM with self-attention. By merging global information with local features, the network more effectively distinguishes target defects from the background, enhancing the detection performance.

After comparison, we observed that in the first set of comparison images, our algorithm is more sensitive to small defects, which makes the network more capable of detecting defects of different scales. In the second set of images, it can be found that in the complex multi-defective scene, our algorithm can accurately detect the defective parts, while the original network cannot detect some of the defects. In the third set of images and the fourth set of images, it is demonstrated that our detection algorithm can improve the detection of small targets and reduce omission detections, achieving higher accuracy in the complex backgrounds.

**Figure 14:**
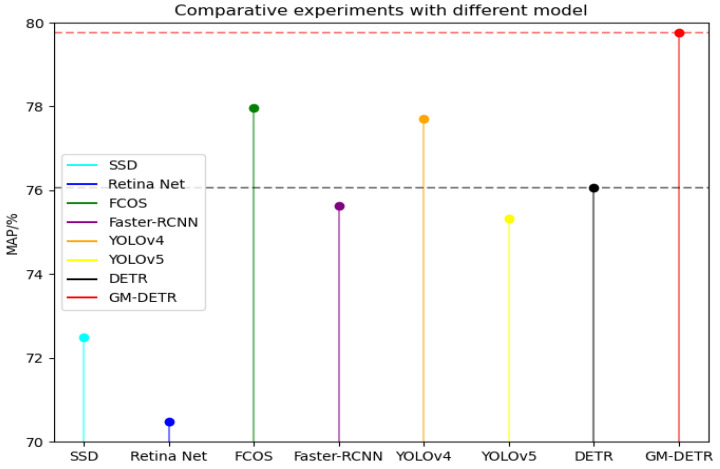
Comparative experiments with different models.

We also compared the detection results of the different networks, including Faster-RCNN [5], YOLOv4 [46], YOLOv5, Retina Net [19], FCOS [20], and SSD [4], as shown in Table 5 and Figure 14. The GM-DETR model has a high accuracy of 79.77 and a good F1-score, as well as a low number of parameters, and performs relatively well in several other aspects. These results demonstrate that GM-DETR can not only improve the problem that the DETR model lacks control of image details due to the complexity of defect types, but also has a good performance on the above images and accuracy. In addition, to avoid the excessive number of model parameters from increasing the computational cost, the layer-pruning strategy proposed in this paper optimizes the decoding layer, which reduces the amount of model parameters by 12.9%. It is further shown that our optimization is further validated about the GM-DETR model.

## 5. Conclusions

In this paper, we address the problem of the DETR model's limitations in controlling image details and its slow convergence, which stem from complex the defective backgrounds and low resolution, by proposing the GM-DETR algorithm. This method uses GAM global attention and self-attention within DETR to combine global information with local features, allowing the network to better distinguish between target defects and background. At the same time, the method recalibrates the local features, reduces the target information dispersion, and amplifies the global feature interactions to improve the performance of the neural network, thus improving the detection performance. Subsequently, to eliminate unnecessary parameters, we implement a layer-pruning strategy, which enhances the computational efficiency of the model. Furthermore, due to the problem of poor sensitivity of the original loss function to defective targets with small differences, this paper chooses to use the MSE loss not only to improve the detection accuracy but also to improve the convergence speed. This method highlights the convenience and simplicity of end-to-end detection compared to traditional methods. The model outperforms other mainstream detection models such as Faster R-CNN, SSD, and Retina Net. The GM-DETR model has higher detection accuracy, lower parameter requirements, and improved model fitting. Specifically, the loss on the validation set decreased from around 0.8 to 0.55, and the small differences between predicted results and actual results confirm the fitting performance of this model in complex scenarios. As a result, the model fits better, improving detection accuracy by 4.9% and reducing the number of parameters by 12.9%. This paper demonstrates that the model we have presented is particularly suitable for scenarios with limited resources, such as edge devices or IoT devices. In future research, our goal will focus on designing an end-to-end defect detection model that addresses a wider range of defect types and can be deployed across a broader range of fields. We aim to enhance the model’s detection capabilities to contribute to the advancement of artificial intelligence in practical applications.

## Figures and Tables

**Table 1:** Experimental environment.

Environmental Parameters	Value
Operating system	Windows11.0
Deep Learning Framework	PyTorch1.11.0
Programming language	Python3.10
CPU	Intel(R) Core(TM) i5-11400F
GPU	GTX 1660 Ti
RAM	16 GB

**Table 2:** Experimental parameters.

Hyper Parameterization	Value
Learning Rate	0.01
Image Size	720 × 720
Momentum	0.9
Optimizer Type	Adamw
Weight Decay	0.001
Batch Size	8
Epoch	300

**Table 3:** Ablation experiments.

GAM	Decoder	Loss	mAP@0.5 (%)	Para (M)	FPS
			76.05	41.00	17.10
√			76.95	43.76	15.20
	√		78.83	35.52	20.78
		√	79.32	41.00	17.60
√		√	77.96	42.34	15.67
√	√	√	79.77	35.71	20.40

**Table 4:** Comparative experiments with different attention.

Models	mAP@0.5 (%)	Para (M)	FPS
DETR	76.05	41.00	17.10
DETR + CA	76.04	41.32	17.02
DETR + SE	76.56	42.20	15.90
DETR + DWR	75.35	41.50	16.73
DETR + ECA	75.92	41.78	17.56
DETR + GAM	76.95	43.76	15.20

**Table 5:** Comparative experiments with different models.

Models	mAP@0.5 (%)	Params (M)	Precision (%)	Recall (%)	F1
SSD	72.50	26.28	88.03	55.98	0.68
FCOS	77.97	36.76	77.91	72.83	0.75
Retina Net	70.47	37.76	90.65	52.72	0.67
Faster-RCNN	75.62	137.09	35.07	80.43	0.49
YOLOv4	77.70	63.50	84.00	74.00	0.78
YOLOv5	75.32	47.92	80.60	58.70	0.68
DETR	76.05	41.00	58.72	80.43	0.68
GM-DETR	79.77	35.71	61.54	82.61	0.71

## Data Availability

The data presented in this study are available on request from the corresponding author.

## References

[1] Peng T., Zheng Y., Zhao L., Zheng E. (2024). Industrial Product Surface Anomaly Detection with Realistic Synthetic Anomalies Based on Defect Map Prediction. Sensors.

[2] Cumbajin E., Rodrigues N., Costa P., Miragaia R., Frazão L., Costa N., Fernández-Caballero A., Carneiro J., Buruberri L.H., Pereira A. (2023). A Real-Time Automated Defect Detection System for Ceramic Pieces Manufacturing Process Based on Computer Vision with Deep Learning. Sensors.

[3] Saberironaghi A., Ren J., El-Gindy M. (2023). Defect detection methods for industrial products using deep learning techniques: A review. Algorithms.

[4] Chen Z., Guo H., Yang J., Jiao H., Feng Z., Chen L., Gao T. (2022). Fast vehicle detection algorithm in traffic scene based on improved SSD. Measurement.

[5] Ren S., He K., Girshick R., Sun J. (2016). Faster R-CNN: Towards real-time object detection with region proposal networks. IEEE Trans. Pattern Anal. Mach. Intell..

[6] Jiang P., Ergu D., Liu F., Cai Y., Ma B. (2022). A Review of Yolo algorithm developments. Procedia Comput. Sci..

[7] Li J., Su Z., Geng J., Yin Y. (2018). Real-time detection of steel strip surface defects based on improved yolo detection network. IFAC-PapersOnLine.

[8] Carion N., Massa F., Synnaeve G., Usunier N., Kirillov A., Zagoruyko S. End-to-end object detection with transformers. Proceedings of the European Conference on Computer Vision.

[9] Cheng Y., Liu D. (2022). An image-based deep learning approach with improved DETR for power line insulator defect detection. J. Sens..

[10] Dang L.M., Wang H., Li Y., Nguyen T.N., Moon H. (2022). DefectTR: End-to-end defect detection for sewage networks using a transformer. Constr. Build. Mater..

[11] Dai X., Chen Y., Yang J., Zhang P., Yuan L., Zhang L. Dynamic detr: End-to-end object detection with dynamic attention. Proceedings of the IEEE/CVF International Conference on Computer Vision.

[12] Zhu M., Kong E. (2024). Multi-Scale Fusion Uncrewed Aerial Vehicle Detection Based on RT-DETR. Electronics.

[13] Czimmermann T., Ciuti G., Milazzo M., Chiurazzi M., Roccella S., Oddo C.M., Dario P. (2020). Visual-based defect detection and classification approaches for industrial applications. Sensors.

[14] Ren Z., Fang F., Yan N., Wu Y. (2022). State of the art in defect detection based on machine vision. Int. J. Precis. Eng. Manuf. Green Technol..

[15] Anitha S., Radha V. Evaluation of defect detection in textile images using Gabor wavelet based independent component analysis and vector quantized principal component analysis. Proceedings of the Fourth International Conference on Signal and Image.

[16] Allili M.S., Baaziz N., Mejri M. (2014). Texture modeling using contourlets and finite mixtures of generalized Gaussian distributions and applications. IEEE Trans. Multimed..

[17] Zalama E., Gómez-García-Bermejo J., Medina R., Llamas J. (2014). Road crack detection using visual features extracted by Gabor filters. Comput.-Aided Civ. Infrastruct. Eng..

[18] Xu Y., Li D., Xie Q., Wu Q., Wang J. (2021). Automatic defect detection and segmentation of tunnel surface using modified Mask R-CNN. Measurement.

[19] Tran V.P., Tran T.S., Lee H.J., Kim K.D., Baek J., Nguyen T.T. (2021). One stage detector (RetinaNet)-based crack detection for asphalt pavements considering pavement distresses and surface objects. J. Civ. Struct. Health Monit..

[20] Yao S., Zhu Q., Zhang T., Cui W., Yan P. (2022). Infrared image small-target detection based on improved FCOS and spatio-temporal features. Electronics.

[21] Xie H., Zhang Y., Wu Z. (2021). An improved fabric defect detection method based on SSD. AATCC J. Res..

[22] Hu B., Wang J. (2020). Detection of PCB surface defects with improved faster-RCNN and feature pyramid network. IEEE Access.

[23] Li Z., Tian X., Liu X., Liu Y., Shi X. (2022). A two-stage industrial defect detection framework based on improved-yolov5 and optimized-inception-resnetv2 models. Appl. Sci..

[24] Cheng X., Yu J. (2020). RetinaNet with difference channel attention and adaptively spatial feature fusion for steel surface defect detection. IEEE Trans. Instrum. Meas..

[25] Wang Y., Zhang X., Yang T., Sun J. Anchor detr: Query design for transformer-based detector. Proceedings of the AAAI Conference on Artificial Intelligence, Carnegie Mellon University.

[26] Li D., Yang P., Zou Y. (2024). Optimizing Insulator Defect Detection with Improved DETR Models. Mathematics.

[27] Wang D., Li Z., Du X., Ma Z., Liu X. (2022). Farmland obstacle detection from the perspective of uavs based on non-local deformable detr. Agriculture.

[28] Han K., Xiao A., Wu E., Guo J., Xu C., Wang Y. (2021). Transformer in transformer. Adv. Neural Inf. Process. Syst..

[29] Karp R.M., Vazirani U.V., Vazirani V.V. An optimal algorithm for on-line bipartite matching. Proceedings of the Twenty-Second Annual ACM Symposium on Theory of Computing.

[30] Mills-Tettey G.A., Stentz A., Dias M.B. (2007). The dynamic hungarian algorithm for the assignment problem with changing costs. Robot. Inst. Pittsburgh.

[31] Vaswani A., Shazeer N., Parmar N., Uszkoreit J., Jones L., Gomez A.N., Kaiser Ł., Polosukhin I. (2017). Attention is all you need. Adv. Neural Inf. Process. Syst..

[32] Ding G., Georgilas I., Plummer A. (2023). A Deep Learning Model with a Self-Attention Mechanism for Leg Joint Angle Estimation across Varied Locomotion Modes. Sensors.

[33] Li J., Wang X., Tu Z., Lyu M.R. (2021). On the diversity of multi-head attention. Neurocomputing.

[34] Li X., Song J., Gao L., Liu X., Huang W., He X., Gan C. Beyond rnns: Positional self-attention with co-attention for video question answering. Proceedings of the AAAI Conference on Artificial Intelligence.

[35] Shao Y., Lin J.C.-W., Srivastava G., Jolfaei A., Guo D., Hu Y. (2021). Self-attention-based conditional random fields latent variables model for sequence labeling. Pattern Recognit. Lett..

[36] Niu Z., Zhong G., Yu H. (2021). A review on the attention mechanism of deep learning. Neurocomputing.

[37] Woo S., Park J., Lee J.-Y., Kweon I.S. Cbam: Convolutional block attention module. Proceedings of the European Conference on Computer Vision (ECCV).

[38] Zhou B., Khosla A., Lapedriza A., Oliva A., Torralba A. Learning deep features for discriminative localization. Proceedings of the IEEE Conference on Computer Vision and Pattern Recognition.

[39] Shi P., Qiu J., Abaxi S.M.D., Wei H., Lo F.P.-W., Yuan W. (2023). Generalist vision foundation models for medical imaging: A case study of segment anything model on zero-shot medical segmentation. Diagnostics.

[40] Zhao H., Gallo O., Frosio I., Kautz J. (2016). Loss functions for image restoration with neural networks. IEEE Trans. Comput. Imaging.

[41] Wang Z., Bovik A.C. (2009). Mean squared error: Love it or leave it? A new look at signal fidelity measures. IEEE Signal Process. Mag..

[42] Hou Q., Zhou D., Feng J. Coordinate attention for efficient mobile network design. Proceedings of the IEEE/CVF Conference on Computer Vision and Pattern Recognition.

[43] Hu J., Shen L., Sun G. Squeeze-and-excitation networks. Proceedings of the IEEE Conference on Computer Vision and Pattern Recognition.

[44] Wei H., Liu X., Xu S., Dai Z., Dai Y., Xu X. DWRSeg: Rethinking Efficient Acquisition of Multi-scale Contextual Inf ormation for Real-time Semantic Segmentation. Proceedings of the Computer Vision and Pattern Recognition.

[45] Wang Q., Wu B., Zhu P., Li P., Zuo W., Hu Q. ECA-Net: Efficient channel attention for deep convolutional neural networks. Proceedings of the IEEE/CVF Conference on Computer Vision and Pattern Recognition.

[46] Hu X., Liu Y., Zhao Z., Liu J., Yang X., Sun C., Chen S., Li B., Zhou C. (2021). Real-time detection of uneaten feed pellets in underwater images for aquaculture using an improved YOLO-V4 network. Comput. Electron. Agric..

